# Dissertation on the Facial Gland

**Published:** 1838-07

**Authors:** 


Art. VIII.—*De Nervo Faciali.* Auctore Doctore Massalien.—*Berolini*, 1836.

*Dissertation on the Facial Nerve.* By Dr. Massalien.—*Berlin*, 1836. 4to. pp. 23.

The author gives a succinct account of the anatomy, functions, and pathology of the facial nerve, chiefly compiled from the researches of Sir Charles Bell on the Nervous System. An interesting case is detailed at full length, of facial paralysis occurring in a scrofulous child from tubercles in the brain. The paralysis was confined to the left side. The patient died hectic and emaciated. On examination after death, three tubercles of various magnitude were found in the cerebral substance. One of them, situated in the cineritious portion of the left hemisphere, was as big as a walnut. The others were of the size of small hen's eggs. On cutting them across, the section presented an external stratified tissue with a radiated core. The former had a bright yellow hue; the colour of the latter was a dingy yellow. The tubercles possessed more than ordinary density. It is a remarkable fact, and one not heretofore demonstrated by microscopic observation, that their structure was composed, not of acini, as is commonly the case, but of fibres. On minute inspection, the mass resembled dried cheese. The two larger tubercles, above mentioned, occupied nearly the middle fossæ of the basis cranii, which are formed by the temporal and occipital bones. They were, in consequence, inserted between the surface of the middle lobes of the brain and the dura mater lining the median fossæ; they were intimately connected with the cortical substance, and invested by the meninges. Of these two tubercles the left deserves most attention, as corresponding with the paralytic side of the face. Of it, the tuberculous matter was evidently traceable into the cerebral lobe; filling the mastoid cells, and passing into the canal of Fallopius; in which latter situation the facial nerve was obviously compressed. The tuberculous substance accompanied the nerve till its exit at the stylo-mastoid foramen. The nerve there seemed somewhat swollen. The external lamina of the petrous bone was necrosed. The membrana tympani was destroyed, and the ossicula auditus, with the exception of the stapes, had perished. The size of the tumours, and their relative position to neighbouring parts, are well shown in an annexed plate of a vertical section of the brain, from a drawing by Dr. R. Froriep.

